# Supplementary material for: Are carbon emissions trading and green financial instruments synergistic? -Comprehensive quantitative research based on content analysis
Source: PLoS One. 2024 Mar 7;19(3):e0298601. doi: 10.1371/journal.pone.0298601 (PMC10919601; doi:10.1371/journal.pone.0298601)
Supplement: S1 Data — (ZIP) [file pone.0298601.s001.zip › Data/地级市Z_F工作报告.zip!/┴╔─■/╜⌡╓▌╩╨/└┤╫╘╣1⁄2╓┌║┼í╛┬φ┐╦╩2╛▌═°í┐_2013.docx]

各位代表：

　　现在，我代表市政府向大会报告工作，请予审议，并请市政协各位委员提出意见和建议。

　　一、过去五年简要回顾和2012年工作情况

　　过去的五年，在省委、省政府和市委的正确领导下，我们紧紧依靠全市人民，全面贯彻落实科学发展观，戮力同心，真抓实干，圆满完成了市十四届人民代表大会确定的各项任务，辽宁沿海第二大城市建设取得突破性进展。

　　经济总量跃上新台阶。全市地区生产总值年均增长14.1%，总量突破千亿元，是2007年的2.4倍；公共财政预算收入年均增长38%，总量是2007年的5倍；累计完成全社会固定资产投资2655.6亿元，是上一个五年的5倍；规模以上工业增加值年均增长16.5%，总量是2007年的3.3倍；社会消费品零售总额年均增长18.6%，总量是2007年的2.3倍。产业结构进一步优化，三次产业结构调整为15.2:49.2:35.6。

　　三大经济区域协调发展。开发区、龙栖湾新区、大有经济区建设全面提速，开发区晋升为国家级开发区，龙栖湾新区晋升为省级开发区。成功申办2013中国·锦州世界园林博览会。锦州港辐射带动作用日益增强，龙栖湾港建设快速推进，光伏、石化、精细化工、汽车及零部件等临港产业不断壮大。县域“双增升位”计划成效显著，“三化”建设同步推进，公共财政预算收入等指标在全省位次大幅提升。城区“三年倍增”计划顺利实施，中央十里商街等服务业集聚区建设全省领先，城区经济和服务业发展活力逐步显现。

　　改革开放实现重大突破。国企改革任务基本完成，公用事业、集体林权、文化体制等改革进展顺利。行政审批制度改革不断深入，审批事项由229项减少到183项。城区区划调整和经济社会管理权限下放工作基本完成。锦州成功获批并成为辽宁唯一的国家加工贸易梯度转移承接地。累计引进外资项目153个，实际利用外资29.2亿美元，年均增长40%；出口创汇59.4亿美元，年均增长23.4 %。累计引进海外研发团队项目23项。锦州银行实现跨区域经营。阳光能源、新华龙钼业等企业成功上市。

　　城乡环境建设日新月异。老城中心区控制性详细规划通过专家评审，锦州湾中国海洋城规划建设全面启动，城市南扩成效显著。锦凌水库主体工程提前一年完成。滨海大道、龙栖湾大道、云飞大桥等建成通车。新建、大修改造城市街路150条。新建市县乡客运站45座,1109个行政村实现村村通油路。8座污水处理厂建成投入使用。燃气、自来水普及率和城市集中供热率分别达到93%、97%和85%。造林绿化185万亩，森林覆盖率达到22%。

　　社会事业取得长足发展。规模以上工业企业高新技术产品增加值年均增长33%，累计完成发明专利申请883件。国家硅材料高新技术产业化基地建设加速推进。各级各类教育加快发展，义务教育全面达到省定“双高”水平 ，职业教育布局调整全省领先。城市医疗“一个中心、多个专科”新格局全面形成，基层医疗卫生机构体系覆盖全市城乡。医药卫生体制改革稳步推进，新型农村合作医疗全面实施。城乡居民健康卡发放工作正式启动。低生育水平持续稳定。“医巫闾山满族剪纸”等非物质文化遗产保护工作卓有成效，京剧《东丹王传奇》等精品剧目获国家及省级大奖。广播电视村村通等惠民工程顺利完成。《锦州文化产业振兴规划纲要》颁布实施，锦州古玩城入选省首批“十强文化企业”。圆满承办了省第七届少数民族传统体育运动会。北京奥运会锦州籍运动员取得2金2银好成绩。

　　人民生活水平明显提高。城镇居民人均可支配收入、农民人均纯收入年均增长分别为13.3%和16.4%。居民消费价格总水平始终控制在全省平均线以下。实名制就业38.9万人，城镇登记失业率低于全省平均水平。社会保障体系日臻完善，基本养老保险体系实现全覆盖。城乡居民最低生活保障基本实现应保尽保。新增养老服务机构18家，农村敬老院发展到81所。建成各类保障性住房26465套，中低收入家庭基本实现住有所居。归集住房公积金43亿元。减少农村贫困人口32.3万人，解决了57.5万农村人口安全饮水问题。食品药品安全、社会稳定和安全生产工作不断加强，平安锦州建设深入推进。

　　各位代表，过去五年，锦州的发展取得了巨大成就，这是省委、省政府和市委正确领导的结果，是全市人民团结奋斗、开拓进取的结果，也是市人大、市政协有效监督、鼎力支持的结果。在此，我代表市政府，向在各个领域和岗位上辛勤劳动、做出贡献的全市人民表示崇高的敬意！向给予政府工作大力支持的人大代表、政协委员、各民主党派、工商联和各人民团体，向关心和支持政府工作的离退休老同志，向中省直各部门、各单位，向驻锦部队和武警官兵，向所有关心支持锦州发展的海内外朋友，表示衷心的感谢！

　　2012年是本届政府任期的最后一年。面对复杂多变的外部宏观经济环境，我们迎难而上，较好地完成了市十四届人大五次会议确定的各项任务。

　　初步核算，全市实现地区生产总值1248.5亿元，增长10.4%；全社会固定资产投资835亿元，增长25%；公共财政预算收入131.5亿元，增长26.9%；规模以上工业增加值590.1亿元，增长11.3%；社会消费品零售总额434.5亿元，增长16%；实际利用外资10亿美元，增长86.7%；引进域外资金770亿元，增长10%；出口创汇17.5亿美元，增长17.1%；城镇居民人均可支配收入22995元，增长14%；农民人均纯收入10788元，增长14.2%。主要经济指标增速均高于全省平均水平，其中实际利用外资增速居全省第1位，公共财政预算收入、社会消费品零售总额增速居全省第2位。锦州巨大的发展潜力日益显现，全市人民热切期盼的强劲发展态势已经形成。

　　（一）沿海经济带建设取得突破性进展

　　全面启动沿海带及锦州湾中国海洋城规划，空间和产业布局不断优化，基础设施建设和产业集群发展步伐加快。

　　世园会筹备建设工作取得决定性胜利。园区基础设施建设全部完工，57个展园基本建成，“一塔三馆”主体工程竣工，市场开发、运营管理工作全面展开。全市上下关心、参与、奉献世园会的氛围日益浓厚，世界的目光开始聚焦锦绣之州、海上世园。

　　港口建设实现重大突破。锦州港完成货物吞吐量7348万吨、集装箱运输85万标箱。3个通用泊位投入试运行，油品罐区一期主体工程完工，中电投煤炭码头正在加快建设。龙栖湾港围堰、防波堤、万吨级航道等工程顺利实施，客货滚装码头前期工作稳步推进。“一港双区”规划纳入国家口岸发展规划。

　　开发区建设迈出新步伐。嘉合化工一期、海森堡石化、石油洞储等一批工业项目和365客服基地、长鸿建国饭店、东一产权式五星级酒店等一批服务业项目开工建设，中储粮仓储及物流、香港宏发东北亚物流基地等项目前期进展顺利。滨海体育中心基本完工。主要街路绿化美化工程高标准完成，笔架山景区、白沙湾浴场展现新形象。

　　龙栖湾新区建设扎实推进。道路管网等基础设施建设不断完善，邰滨支线8.5公里一级路竣工通车。十里金沙滩、景观绿化等世园背景区建设全面展开。客运中心建设加快推进，光伏大厦、公安大厦竣工。奥克阳光多晶硅片、旭龙太阳能电池、世界窗电子、差别化纤维一期20万吨等项目进展顺利。

　　锦州湾机场立项获批。航空物流城主干路和飞行区地基处理工程开工建设。

　　大有经济区建设全面启动。空间规划、产业发展规划编制完成，起步区基本实现“七通一平”。凌海大道建成通车。佐源菊苣糖、仁禾生姜深加工、天沐温泉度假等5个10亿元以上项目开工建设。

　　（二）工业经济运行质量稳步提升

　　深入实施五项工程，依靠科技进步，狠抓生产要素保障，工业经济实现企稳回升。新增亿元以上工业项目150个，其中5亿元以上15个。规模以上企业达到777户。

　　锦州钛业3万吨氯化法钛白粉、石化公司130万吨加氢裂化装置、中信金属中低碳锰铁扩能改造等项目竣工投产。宝钛华神4万吨固体废料综合利用、百威英博一期30万吨啤酒等项目加快推进。锦州钛业6万吨氯化法钛白粉扩能、香港最胜一期100万吨镍合金等项目开工建设。华润电力项目获批。嘉合化工二期、奥鸿药业医药园等项目前期进展顺利。

　　金城纸业恢复上市，步入正常生产运营。

　　光伏、钛及特种金属、石化及精细化工、汽车及零部件、义县新型冶金材料等5个省重点产业集群产值超1000亿元。国家级光伏材料质量监督检验中心、锦州光伏公共研发和服务平台建成投入使用。

　　国家科技成果转化服务（锦州）示范基地获批挂牌。攻克重大关键技术78项，开发新产品580种，有效发明专利达到299件。4户企业被认定为省级技术中心。引进外国专家项目10个，完成海外并购项目5个。规模以上工业企业高新技术产品增加值增长37%。

　　（三）县域经济发展势头强劲

　　四县（市）完成地区生产总值653.6亿元，增长10.3%；固定资产投资400.2亿元，增长29.1%；公共财政预算收入57.3亿元，增长31.5%。县域综合实力在全省排位进一步提升。

　　工业园区及产业集群建设取得新进展。完成基础设施建设投入10.5亿元，新增标准厂房6.7万平方米，建成污水处理厂、垃圾焚化处理场6座。北镇工业炉及配套、义县新型冶金材料两个产业集群初具规模，联众重工、天晟重工、垚鑫铸业、鑫泽锰业等一批亿元以上项目开工建设。黑山、凌海主导产业集群基本确立。义县七里河、黑山庞河产业园区晋升为省级园区。

　　农业综合生产能力大幅提升。粮食总产量达到54.5亿斤，再创历史新高。设施农业面积达到183万亩。蔬菜、水果、肉蛋奶、水产品总产量分别达到385万吨、96万吨、157万吨、42万吨。黑山中德肉禽、北镇金源葡萄酒、凌海裕海海珍品、义县双双食品等8个10亿元以上农产品加工项目开工建设。农业产业化龙头企业达到498家。“一县一业”基本确立，黑山、北镇成为省示范县。

　　县域城镇化进程不断加快。四县（市）县城规划通过省审批。完成房地产开发面积300万平方米，城镇化率提高2个百分点。

　　城乡建设用地增减挂钩、盘活存量土地、耕地保护、节约集约用地工作卓有成效。全年建设用地规模超10平方公里，是近几年最多的一年。

　　农村生产生活条件进一步改善。新增节水滴灌面积12.1万亩。完成大小凌河封育滩地治理18万亩、河道生态治理10.6万亩、小流域治理43.9万亩。青山工程全面实施，绿化造林32万亩。气象防灾减灾服务能力明显增强。耕种收综合机械化水平达到69%。免疫无口蹄疫区通过国家认证，重大动物疫病和畜产品安全事件“零发生”。新建、大中修农村公路959公里。建成锦凌水库移民新村9处。

　　（四）城区经济和服务业发展步伐加快

　　推进城区区划调整，下放经济社会管理权限51项，激发了城区发展活力。城区完成地区生产总值532.1亿元，增长10.4%；固定资产投资403.5亿元，增长22.3%；公共财政预算收入57.1亿元，增长29.6%。

　　12个现代服务业集聚区列入省指标统计体系。亿隆国际、温州名购、国际五金机电城等一批在建项目快速推进，二轻国税、原铁路医院、松山薛西等地块商业开发项目前期进展顺利。

　　房地产开发投资、商品房销售额分别增长20%和15%。

　　《锦州市旅游发展总体规划》编制完成，“三带、九区、五镇、四十八景”项目建设全面启动。5个温泉项目开工建设，其中凌海九华山温泉项目建成投入运营。全市旅游人数突破2000万人次，旅游业收入增长55.4%。成功举办了农博会、古玩节、房交会、汽车博览会等展会。

　　投融资工作取得历史性突破。优化资源配置，做大做强城投、华信、北发、龙信等投融资公司。启动了中期票据、信托、城投债、企业债等融资工作。全市新增贷款125亿元，发行城投债券13亿元，BT融资6.6亿元。破解了锦凌水库贷款难题。新组建小额贷款公司11家、融资担保公司1家，光大银行锦州分行挂牌营业。

　　（五）对外开放成效日益凸显

　　创新招商引资体制机制，组建了长春、天津、宁波、深圳等4个驻外招商分局，对外招商活动日趋活跃。参加4次省政府境内外招商活动，自行组织20余次，海洋城、十里商街、县城建设等主题概念招商影响广泛，收效明显。365客服基地、百脑汇IT总部大厦等一批项目落户锦州。

　　积极推进与世界500强企业合作。与中国化工、中国五矿、华润、中国建材、新兴际华、中船工业、中信重工、印尼力宝、韩国SK等国内外知名企业达成合作意向。开发区美国胡商集团总部基地及市场物流园、北镇国测石墨科技产业园等一批重大项目成功签约。

　　加大对重点出口企业扶持力度，千方百计开拓国际市场。石化、汽车及零部件、特种铁合金等特色产业出口基地加快建设。锦州获批省内首个新能源类“国家科技兴贸创新基地”。

　　（六）城市规划和基础设施建设水平明显提高

　　坚持规划先行。锦州沿海带概念规划、锦州湾中国海洋城总体规划、十里商街控详规划、城市综合交通体系规划、城市水生态环境综合治理规划编制完成。城市规划建设的基础工作得到加强，为城市的未来发展提供了有力支撑。

　　锦凌水库主体工程基本竣工。城市水系建设工程一期、解放路东联、滨海大道东联工程开工建设。中央南街立交桥、广州街公铁立交桥工程加快推进。新建改造城区街路53条。京沈高速公路东出口拓宽改造等10项迎世园重点交通工程基本完工。

　　强力推进市容环境综合整治工作。理顺城市管理体制，街路管理标准提档升级，市容环境有所改观。400万平方米非物业小区整治年度任务基本完成。士英街等5条街路绿化工程全部完成，5个城市出入口绿化改造工程全面启动。

　　八角台城市供水工程竣工，有效缓解了城市供水压力。

　　新建污水处理厂12座，拆除并网供热小锅炉17台，完成供热计量及节能改造100万平方米。大、小凌河干流及其主要支流水质全部达到四类标准。城市空气质量优良率达到96.7%。

　　（七）民生工程和社会事业取得新成就

　　实名制就业8.8万人，城镇登记失业率2.7%。超额完成“五大保险”省定指标。城乡低保对象救助和农村五保对象供养标准不断提高，“一站式”、“普惠制”医疗救助模式全面推行。4所农村病人托管中心、15所社区老人服务中心建成投入使用。建设完成保障性住房4616套。11.2万农村低收入群众年人均纯收入超过2200元。慈善事业进一步发展，爱心救助活动成效明显。

　　教育综合实力全面提升。职教园二期工程即将完工。新建改造危房校舍3.3万平方米，改扩建幼儿园27所。荣获“省级优秀卫生城市”殊荣，率先在全省建立区域卫生信息平台，作为全国试点城市首发居民健康卡。发放计划生育家庭特别扶助金和奖励扶助金3816万元，计划生育家庭保障水平明显提高。

　　博物馆、纪念馆、图书馆、艺术馆向市民免费开放。成功举办首届全民读书节。木偶新剧《渔夫与金鱼》荣获国际木偶节“优秀节目奖”。义县奉国寺大雄殿入选《中国世界文化遗产预备名单》。数字电视整体转换工作稳步推进。新华书店、广播电视报社等转企改制工作基本完成。圆满承办了中国女子篮球甲级联赛、全国羽毛球锦标赛等国家级体育赛事。首届全民健身体育运动会取得圆满成功。

　　城区、农村警务改革工作全省领先，公安信息化和社会治安防控体系建设不断加强，打击各类犯罪、维护社会稳定更加有力，群众安全感进一步增强。“六五”普法顺利实施，乡镇、街道法律援助工作站（点）覆盖率达到100%。“农超对接”菜篮子工程正式启动。物价监管工作成效明显。食品药品安全、应急管理、信访稳定、安全生产等工作全面加强。

　　（八）民主法制和政府自身建设进一步加强

　　自觉接受人大法律监督、政协民主监督和群众的社会监督，坚持定期向人大和政协报告、通报工作，积极采纳各民主党派、工商联、无党派人士意见。全年共办理市人大代表建议和政协委员提案333件，办复率、见面率均达100%。

　　全面推行依法行政，行政监察、审计监督力度不断加大，政务公开进一步向基层延伸，行政复议作用更加明显。积极推动惩治和预防腐败体系建设，政府机关勤政廉政意识不断增强。

　　第五次行政审批制度改革圆满完成，审批程序逐步规范，审批效率明显提高。不断强化政府绩效考评机制，卫生、林业、安全生产等省政府绩效考评指标有望跻身全省前三位。

　　切实转变工作作风，不断增强各级干部勇于担当、敢于负责的责任感和使命感，不断激发干部群众的工作激情，政府系统应对复杂局面、破解发展难题的能力不断提升。

　　国防动员、边海防建设、人防和民兵预备役工作取得了新进步，拥军优属、军民共建活动深入开展，全省首家军粮直供示范店开业。民族团结进步创建活动扎实推进。国家安全、妇女儿童、外事侨务、防震减灾和地方志工作不断加强。统计、质监、工商、贸促、残联等工作取得新进展。

　　在看到成绩的同时，我们也清醒地认识到，发展中还面临着不少困难和问题。主要是：经济总量偏小，结构性矛盾还比较突出；地区间发展不平衡，沿海开发建设还相对滞后；具有支撑力的重大项目还不多；城乡居民收入分配差距依然较大，部分群众生活还很困难；维护社会稳定、创新社会管理的任务还十分艰巨；民主法制和政府自身建设有待进一步加强。对于这些问题，我们要高度重视，采取有力措施，切实加以解决。

　　二、今后五年工作展望和2013年工作安排

　　今后五年，是锦州经济社会发展的重要战略机遇期，也是建设辽宁沿海第二大城市、率先全面建成小康社会的关键时期。任务艰巨，时不我待。我们要全面贯彻落实党的十八大精神，贯彻落实省委、省政府的决策部署，以科学发展为主题，以加快转变发展方式为主线，以提高经济质量和效益为中心，深入实施沿海突破战略，不断深化改革开放，坚持增量带动结构优化、创新促进产业升级、发展保障改善民生，全面加快辽宁沿海第二大城市建设步伐，力争早日全面建成小康社会。

　　全市经济社会发展目标是：经过三年努力，到2015年末，力争地区生产总值和城乡居民人均收入比2010年翻一番，三次产业结构调整为10:50:40，全面完成“十二五”规划的目标任务;再经过两年奋斗，到2017年末，力争地区生产总值突破3000亿元，公共财政预算收入突破300亿元，城镇居民人均可支配收入和农民人均纯收入分别达到44800元和20000元，文化、教育、科技、卫生等社会事业和社会保障都有长足的进步，把锦州建设成为辽西中心城市、辽宁沿海第二大城市，提前全面建成小康社会。

　　按照上述要求，在实际工作中，我们要突出以下重点：

　　——坚持统筹兼顾、“四化”同步，努力实现科学发展。坚持走新型工业化、信息化、城镇化、农业现代化道路，统筹推进沿海、县域、城区三大经济区域协调发展，开创经济运行高效、经济增长协调、经济结构优化、经济社会和谐的科学发展局面。

　　——坚持深化改革、扩大开放，努力实现率先发展。紧紧咬定提前全面建成小康社会奋斗目标，切实增强率先发展的使命感和紧迫感，进一步深化改革，全方位扩大开放，通过五年的不懈努力，实现锦州在辽西领先、在全省发展位次前移。

　　——坚持人才优先、科技支撑，努力实现创新发展。发挥我市科技、教育、人才资源优势，优化创新资源配置和综合利用，让一切劳动、知识、技术、管理和资本活力竞相迸发，让一切创造社会财富的源泉充分涌流，努力使创新驱动成为锦州发展进步的重要推动力量。

　　——坚持规划先行、改善生态，努力实现绿色发展。坚持以规划引领建设、推动发展，完善城市功能，提升城市品位。加速城乡一体化进程，把锦州建设成为城市建成区200平方公里、城市人口150万人的特大型城市。扎实推进生态文明建设，努力使锦州的天更蓝、山更绿、水更清。

　　——坚持以人为本、民生为重，努力实现和谐发展。坚持城乡居民收入增长高于经济发展增速。创新社会管理，注重社会建设，着力保障和改善民生。推进和谐锦州建设进程，努力使改革发展成果更多地惠及全市人民。

　　各位代表，2013年是全面贯彻落实党的十八大精神的开局之年，也是新一届政府的起步之年。我们要按照中央、省经济工作会议和市委十一届四次全会精神，突出重点、稳中求进，开拓创新、扎实开局。

　　经济社会发展预期目标是：地区生产总值增长10%；全社会固定资产投资增长25%；公共财政预算收入增长15%；规模以上工业增加值增长12%；社会消费品零售总额增长16%；实际利用外资、引进域外资金和出口创汇分别增长10%；城镇居民人均可支配收入增长10%，农民人均纯收入增长12%。在实际工作中，我们将朝更高的目标努力，力争更好更快发展，确保首季开门红，半年双过半，全年主要指标增速不低于全省平均水平、总量不低于去年在全省的位次。

　　按照以上目标和要求，我们要重点抓好九个方面的工作：

　　（一）加快基础设施建设，全面提升未来发展的支撑力、竞争力

　　全面完成锦凌水库建设。确保水库如期竣工蓄水。高标准编制库区总体发展规划，切实做好水土保持及环境保护工作。完成移民安置工作。大力推进以小凌河、女儿河、百股河整治为重点的水系建设，完成城市上游一期工程，开工建设城市段二期工程。

　　加快港口建设。完成锦州港航道拓宽、西防波堤、中丝集团油罐区、中能国际褐煤仓储等工程建设，确保中电投煤炭码头主体工程完工，实现港口吞吐量8500万吨、集装箱运输90万标箱。推进龙栖湾港各项基础工程建设，实现简易通航，客货滚装码头投入使用。启动实施龙栖湾港疏港公路、铁路和物流园区建设。

　　全面启动锦州湾机场建设。完成跑道基础工程和航站楼建设，确保明年上半年投入使用。完成航空物流城、通用航空产业园周边道路工程建设，确保锦州湾机场南北两线进出顺畅。

　　滨海体育中心竣工投入使用。

　　加强道路建设。新建城区道路10条、大修改造57条。开工建设中环西路、广州街跨小凌河大桥工程，启动士英街跨沈山铁路高架桥、人民街跨小凌河大桥前期工作，中央南街立交桥竣工通车。充分论证，科学决策，适时启动城市轨道交通建设。

　　加快解放路东联、滨海大道东联及跨大凌河特大桥建设。启动黑山至沈阳高速公路、右滨支线、邰屯至南站一级路前期工作。确保阜盘高速锦州段60公里竣工通车。

　　加强公用设施建设。完成3座热源厂和3项气化工程建设。开工建设日供水25万吨的锦凌水库净水厂。完成百股河截污干管工程。启动污水处理厂中水回用管线铺设工程。

　　（二）全力办好世园盛会，全面提升沿海开发建设水平

　　举全市之力办好世园会。完善园区各项工程及配套服务设施建设，完成背景区及各项服务保障设施建设。全面启动市场开发工作，强化园区安保和管理运营。力争开通8条铁路旅游专线及游轮航线、国际航空支线，确保1000万人入园参观。策划组织好2013世界海洋日暨中国海洋宣传日等重大活动，向世人呈现一届安全、和谐、生动、精彩的世园盛会。同时，在全市广泛组织开展“当好东道主、办好世园会”系列活动，向世人展示锦州的城市风采和文明风尚。

　　全力推进沿海公共基础配套设施和重大项目建设。加快推进省农经校、厚德商贸城等10个在建项目建设，启动市行政服务中心、辽宁医学院、锦州中学整体搬迁等10项工程建设，确保客运中心、安保指挥中心投入使用。整体搬迁改造6个村屯，全面完善道路管网等配套设施，实现沿海带整体环境的提档升级。

　　全力推进临港重大产业项目建设。力争中国化工炼化一体化项目落户开发区；确保开发区嘉合化工一期、中北石化60万吨润滑油等7个化工项目投产达效；龙栖湾世界窗电子、奥克阳光、差别化纤维一期等项目建成投产；大有精细化工产业园引进亿元以上项目10个，实现产业集聚发展，壮大沿海石化轻纺、光伏、精细化工等产业集群规模。

　　全力推进滨海服务业发展。加快365客服基地、天沐温泉度假、大有温泉生态城等项目建设，确保长鸿建国饭店、十里金沙滩、游艇码头等项目世园会期间投入运营，启动白沙湾温泉休闲度假区、五矿休闲旅游小镇、国际航空俱乐部、游艇俱乐部、国家帆板基地、健康谷医学城和西部控股智慧港城基地等项目建设。加快形成以世博园为中心的滨海旅游休闲产业带。用项目建设聚集人气、扩大对外影响。

　　（三）实施工业五项工程，全面提升工业经济运行质量

　　全力抓好项目工程。全年安排千万元以上工业项目700个，其中亿元以上143个、10亿元以上22个。完成工业固定资产投资500亿元，增长22%。确保香港最胜一期100万吨镍合金等8个重大项目竣工投产；华润电力等7个重大项目开工建设；力争黑山煤化工、北镇石墨、凌海大有精细化工、义县石材、开发区国家级炼化一体化基地、汤河子精铁矿粉深加工和龙栖湾纺织、通用航空、特种船舶制造等9个在谈重大项目早日落地。

　　全力抓好产业集群工程。不断壮大光伏、钛及特种金属、石化及精细化工、汽车及零部件、义县新型冶金材料等5个省级重点产业集群。加快推进“分布式”光伏电站、旭龙太阳能30兆瓦非晶薄膜电池、奥克阳光500兆瓦多晶硅片、北镇100兆瓦农业光伏等项目建设，积极创建国家级新能源示范市，坚定不移地推动“光伏之都”建设；依托锦州石化年产25万吨烷基化装置等项目，加快推进精细化工产业基地建设；依托锦州钛业、宝钛华神等项目，拉长钛系列产品产业链条，打造“中国钛都”。大力扶持沟帮子工业炉及配套等5个市级产业集群，积极培育义县石材等5个新兴产业集群。

　　实施企业提升工程。新增规模以上工业企业50户、销售收入10亿元以上企业4户，力争形成新的销售收入超百亿元企业。

　　全力抓好并购工程，完成企业并购项目5个。

　　全力抓好淘汰落后产能工程，完成省下达的任务指标。

　　加快企业技术创新平台建设。加强与高校、科研院所的合作，启动创新型企业示范工程，确保有效发明专利拥有量增长15%，规模以上企业高新技术产品增加值增长30%以上。优化各类生产要素配置，加快推动工业地产发展，切实帮助企业开拓市场，确保工业经济稳定运行。

　　（四）坚持“三化”同步推进，全面提升县域经济实力

　　咬定“五年双倍增”目标，推进县域工业化、城镇化和农业现代化。确保县域地区生产总值增长15%、公共财政预算收入增长30％、固定资产投资增长40%，经济总量、公共财政预算收入在全省排位明显前移。

　　进一步完善县域工业园区基础设施建设，确保每个县（市）新增建成区面积2平方公里、标准化厂房5万平方米，初步建成检测、研发、金融等公共服务平台。新上固投亿元以上工业项目20个，其中10亿元以上2个。义县新型冶金材料、北镇工业炉及配套两个产业集群分别实现销售收入200亿元和100亿元。黑山、凌海要进一步明确主导产业集群，加快项目引进建设，尽快形成产业规模。

　　推动现代农业发展，积极创建“国家级现代农业示范市”。加快黑山蛋鸡、北镇葡萄、凌海小菜、义县乳业等“一县一业”建设，培育壮大绿色芳山、百通食品等33个国家和省级重点龙头企业，力争每个县（市）再引进1—2个10亿元以上农产品加工项目。

　　加快黑山“两城一区”、义县宜州新城、凌海凌河新城和北镇广宁新区、沟帮子河西新区等新城新市镇建设，高标准规划建设中心商业街，稳步推进农村初中进县城，力争完成县域房地产开发面积500万平方米。

　　实施畜禽标准化、规模化养殖，进一步加强动物疫病防控。保障发展用地，推进节约集约用地。新增设施农业面积10万亩、节水滴灌20万亩，治理水土流失27万亩。不断提高气象防灾减灾应急处置能力。耕种收综合机械化水平达到71%。推进102国道北镇东环段续建和阜锦线义县县城段公路改建工程，完成365公里农村公路黑色路面工程。

　　（五）加快服务业集聚区建设，全面提升城区经济发展水平

　　深入实施“三年倍增”计划，巩固管理权限下放成果，全面激发城区发展活力，确保城区地区生产总值增长10%、公共财政预算收入增长26%、固定资产投资增长30%。

　　全力打造以十里商街为重点的现代服务业集聚区。依据十里商街——锦州金谷控制性详细规划，加快启动拆迁改造建设，确保亿隆国际、温州名购、国际五金机电城等10个项目建成运营，抓紧推进百脑汇IT总部大厦、太阳广场、华润购物广场、红星美凯龙家居广场等10个项目开工建设，力促凤凰卫视、台湾远东、印尼力宝等10个在谈项目早日落地。

　　全面繁荣城乡消费市场。大力发展电子商务、研发设计等现代服务业，精心办好农博会、古玩节等精品展会。全力推进旅游“三带、九区、五镇、四十八景”项目建设，加快温泉旅游开发，大力发展沟域旅游，力争全市旅游人数突破3000万人次。

　　实现投融资工作新突破。整合资源，挖掘潜力，进一步增强华信、城投、北发、龙信等公司的投融资能力，全面做好中期票据、信托、企业债、城投债发行工作，确保市本级直接融资实现重大突破，为基础设施建设、生态环境建设提供强大的资金支持。

　　积极推进招商银行、浦发银行在锦设立分支机构，确保银行新增贷款增长20%。力争锦州银行、五峰米业、锦州钛业3户企业成功上市。

　　确保房地产开发投资和商品房销售额分别增长15%以上。扎实做好服务业一套表联网直报工作。

　　（六）加大招商引资力度，全面提升对外开放水平

　　抢抓国内外资本、产业转移重大机遇，夯实项目承载基础，突出主题概念招商，着力提高外资利用质量和水平。

　　实施重大招商项目攻坚计划。围绕工业产业集群、服务业集聚区、县城建设，开展多层次、多主题的专业化招商，重点突出长三角、珠三角及香港、台湾等地区主题概念招商，力争有更多世界500强、国内500强和行业领军企业落户锦州。建立完善“领导联系制度”和“绿色通道制度”，确保在谈项目尽早签约、签约项目早日开工建设。

　　充分利用国家促进外贸增长政策，加强对锦州石化、中信金属、东方集装箱等20户重点出口企业的跟踪服务，推进特色出口基地建设，支持中小企业开拓国际市场，确保外贸出口稳定增长。

　　积极推进具备条件的经济园区申报国家保税区或晋升省级经济园区。加强科技创新体系建设，力争松山高新区晋升为国家级高新区。确保引进海外研发团队10个以上。

　　（七）强化规划引领作用，全面提升城市建设管理运营水平

　　加快城市总体规划修编，完成城市地下空间开发利用和城市人防工程建设规划，完善城市交通体系、城市生态环境等专项规划和十里商街、城市滨河景观带等重要空间节点规划，进一步做优城市发展空间。

　　提高城市建设标准。严格城市重要节点、滨海重要空间规划和建筑设计方案的审批，力争打造一批城市标志性建筑，进一步提升城市品味、功能价值和整体形象。

　　巩固市容环境综合整治成果。取缔城区人力客运三轮车，拆除有碍观瞻的广告牌匾，逐步实现垃圾分类收集处理和街路机械化清扫。完成城区5个出入口绿化美化亮化，完成剩余400万平方米非物业小区综合整治，完成主要街路360栋建筑平改坡及立面粉饰工程。建立健全城市管理长效机制，强化督察，严格奖惩，切实解决好群众反映强烈的脏、乱、差问题，以崭新的城市面貌迎接世园会召开。

　　进一步提升生态环境建设水平。继续推进凌河保护、辽河治理工程，全面完成年度任务。切实抓好青山工程，确保新增造林绿化100万亩，森林覆盖率提高2个百分点以上。新建卫生填埋场1座。关闭南山、女儿河、百股河水源地。确保12座新建城市污水处理厂稳定运行、城市医疗垃圾全部集中处理、出市境4条河流断面考核全部达标。城市空气质量优良率达到95%以上。

　　（八）着力保障改善民生，全面提升社会事业发展水平

　　坚持以创业带动就业，积极促进高校毕业生、零就业家庭、棚户区回迁居民等重点群体稳定就业。继续扩大各项社会保险覆盖面。全额资助城乡困难群众参加城镇居民基本医疗保险和新型农村合作医疗，实现城乡困难群众救助一体化。进一步做好保障性住房建设和分配工作。切实解决社区200平方米以下办公用房问题。建设县级养老服务中心2个，新增养老床位800张。解决8.6万农村人口安全饮水问题，确保9.8万农村低收入群众年人均纯收入达到2500元以上。

　　推进基础教育强县（市）区建设。大力发展职业教育。规范发展学前教育，新建改建乡镇中心幼儿园、村办园35所。确保校车校园安全。深化医疗卫生体制改革，巩固完善基本药物制度，引导和鼓励社会资本进入医疗领域。加快卫生信息化建设，完成居民健康卡发放，实施健康云服务工程，着力打造健康城市。继续落实计划生育家庭奖励扶助和特别扶助政策，全面开展农村计划生育免费优生健康检查工作。健全残疾人社会保障和服务体系，切实保障残疾人权益。

　　全力做好世园会文化演艺工作，推进世博园省级文化产业示范园区建设。整合市直国有文艺院团资源，规划建设辽西大剧院。启动奉国寺世界文化遗产公园建设，加强广济寺、万佛堂石窟等古建筑维修保护工作。建设抗日义勇军与国歌发祥地纪念馆。深入开展送戏下乡等文化惠民工程。贯彻落实《全民健身计划条例》，广泛开展群众性体育活动。高标准承办好第十二届全运会锦州赛区赛事。

　　不断创新社会管理，全面加强公安基层基础设施和队伍建设，突出做好世园会、全运会安保工作，进一步提升维护公共安全和社会稳定能力。稳妥推进事业单位分类改革。认真开展好第三次全国经济普查。扎实推进“六五”普法，加强基层司法所建设。完善信访长效机制，及时有效化解各类社会矛盾。继续抓好物价监管、食品药品安全、安全生产等工作，全力维护锦州社会稳定。

　　（九）进一步加强自身建设，全面提升政府行政效能

　　自觉接受监督，科学民主决策。认真接受人大法律监督、工作监督和政协民主监督，坚持重大事项向人大报告和向政协通报制度，坚决执行人大及其常委会的各项决议、决定，积极支持人民政协履行职能，认真办理人大议案、代表建议和政协提案。

　　规范行政行为，提高行政效能。加大行政监察、审计监督、政务督察力度，推进政务公开向基层延伸，完善政绩考核机制，进一步规范各级政府和公务员行政行为。巩固第五次行政审批制度改革成果，大力提升公共服务水平。强化廉洁自律意识，继续推进“五大系统”建设，努力建设廉洁高效、人民满意的服务型政府。

　　切实转变作风，增强发展能力。严格落实中央和省有关规定，坚持深入基层、深入实际，真正帮助基层、帮助群众解决实际困难和问题。雷厉风行，只争朝夕，进一步提高政府系统的工作效率。加强专业技能培养，勇于担当、敢于负责，全面提高领导科学发展、破解发展难题的能力。

　　各位代表，新的形势催人奋进，新的征程任重道远，任何艰难险阻都阻挡不住我们前进的步伐。让我们在党的十八大精神指引下，在市委的坚强领导下，紧紧团结依靠全市人民，戮力同心，奋勇拼搏，为加快推进辽宁沿海第二大城市建设，为开创锦州更加幸福美好的明天而努力奋斗！
